# Spatio-temporal mapping of leaf area index in rice: spectral indices and multi-scale texture comparison derived from different sensors

**DOI:** 10.3389/fpls.2024.1445490

**Published:** 2024-09-06

**Authors:** Changming Li, Xing Teng, Yong Tan, Yong Zhang, Hongchen Zhang, Dan Xiao, Shanjun Luo

**Affiliations:** ^1^ Engineering Technology Research and Development Center, Changchun Guanghua University, Changchun, China; ^2^ Rural Energy and Ecological Research Institute, Jilin Academy of Agricultural Sciences, Changchun, China; ^3^ School of Physics, Changchun University of Science and Technology, Changchun, China; ^4^ School of Electrical and Information Engineering, Changchun Guanghua University, Changchun, China; ^5^ Aerospace Information Research Institute, Henan Academy of Sciences, Zhengzhou, China

**Keywords:** leaf area index, UAV, RGB images, multispectral images, texture, machine learning

## Abstract

**Introduction:**

Monitoring the leaf area index (LAI), which is directly related to the growth status of rice, helps to optimize and meet the crop’s fertilizer requirements for achieving high quality, high yield, and environmental sustainability. The remote sensing technology of the unmanned aerial vehicle (UAV) has great potential in precision monitoring applications in agriculture due to its efficient, nondestructive, and rapid characteristics. The spectral information currently widely used is susceptible to the influence of factors such as soil background and canopy structure, leading to low accuracy in estimating the LAI in rice.

**Methods:**

In this paper, the RGB and multispectral images of the critical period were acquired through rice field experiments. Based on the remote sensing images above, the spectral indices and texture information of the rice canopy were extracted. Furthermore, the texture information of various images at multiple scales was acquired through resampling, which was utilized to assess the estimation capacity of LAI.

**Results and discussion:**

The results showed that the spectral indices (SI) based on RGB and multispectral imagery saturated in the middle and late stages of rice, leading to low accuracy in estimating LAI. Moreover, multiscale texture analysis revealed that the texture of multispectral images derived from the 680 nm band is less affected by resolution, whereas the texture of RGB images is resolution dependent. The fusion of spectral and texture features using random forest and multiple stepwise regression algorithms revealed that the highest accuracy in estimating LAI can be achieved based on SI and texture features (0.48 m) from multispectral imagery. This approach yielded excellent prediction results for both high and low LAI values. With the gradual improvement of satellite image resolution, the results of this study are expected to enable accurate monitoring of rice LAI on a large scale.

## 1 Introduction

Rice (Oryza sativa L.) is one of the most important food crops in the world and plays a vital role in food security and socio-economic development globally, especially in Asia (Zhang et al., 2023). Leaf Area Index (LAI) is an essential parameter in ecology and agricultural science, defined as the total leaf area of plants per unit of ground area (Sun et al., 2023). LAI plays a crucial role in understanding the functioning of plant communities, the carbon cycle of ecosystems, the hydrological cycle, and the accuracy of climate models (Yuan et al., 2023). In agriculture, LAI monitoring helps guide crop management practices such as irrigation, fertilization, and pest control. Proper LAI contributes to higher crop yield and quality while reducing resource wastage (Che et al., 2023).

Traditionally, LAI was accessed mainly through manual destructive sampling. This method is time-consuming, labor-intensive, and causes irreversible damage to plants, making it difficult to apply for monitoring crop growth over large areas (Jay et al., 2019). Numerous studies have also been conducted on the inversion of crop LAI through physical modeling. Although the physical meaning of this method is clear, it involves many input parameters, a complicated process, and the problem of “pathological inversion”, which is not convenient for practical application (Zhao et al., 2023). Remote sensing technology, with its advantages of low cost, large coverage area, rapid data acquisition, and dynamic monitoring capabilities, offers the possibility of non-destructive and precise monitoring of crop growth (Lee et al., 2023). Unmanned Aerial Vehicles (UAVs) can capture various types of images via low-altitude flights, making them well-suited for agricultural applications. They effectively address the limitations of satellites and ground-based platforms, offering a valuable tool for precision agriculture (Osco et al., 2021). UAVs are not affected by cloud cover and provide greater flexibility in terms of temporal resolution. In addition, due to the advantages of low hardware cost, high flexibility, ease of operation, and high spatial and temporal resolution of the acquired images, UAVs provide a new technological tool for extracting crop growth information in the field in a fast and non-destructive manner (Luo et al., 2022a, b).

Various sensors carried by drones are utilized in different scenarios. Hyperspectral cameras, for example, are frequently used for the estimation of LAI and chlorophyll content (Cheng et al., 2022; Xie et al., 2014). Thermal infrared and LiDAR sensors are employed to measure vegetation canopy temperature and water content, as well as to estimate biomass and yield (Andújar et al., 2013; Noguera et al., 2020). However, these sensors are heavy for UAVs, expensive, and the acquired data is challenging to process and analyze. Relatively inexpensive RGB cameras can acquire ultra-high-resolution visible light images and are commonly practiced in agriculture as well (Li et al., 2019). Additionally, multispectral cameras can be applied to acquire remote sensing data at high spatial resolution (centimeter level) across multiple wavelengths (from visible to near-infrared) and can strike a balance between cost and availability (Deng et al., 2018). Therefore, RGB and multispectral cameras were selected for the comparative analysis in this study.

In recent years, a large number of applications based on spectroscopic principles have emerged for analyzing the absorption and reflection of electromagnetic spectra by different crop canopy components, and thus constructing vegetation indices (VIs) to monitor crop growth (Lu et al., 2020). Vegetation canopy spectra are closely related to vegetation growth since the plant canopy reflectance carries valuable information about the interaction of the canopy with solar radiation, including absorption and scattering by the vegetation (Derraz et al., 2023). Moreover, on the basis of spectral information, different textures have been proposed to assist in the extraction of growth parameters such as crop LAI, above-ground biomass, and canopy chlorophyll content (Hlatshwayo et al., 2019; Qiao et al., 2020; Zhang et al., 2022). For example, the gray-level covariance matrix (GLCM) was adopted to characterize the distribution of wheat and soil at different stages, which enhanced the accuracy of estimating aboveground biomass of wheat and addressed the problem of underestimating biomass at later stages (Yue et al., 2019). Fourier texture was exploited to simulate the growth trend of rice and to identify the characteristics of monopoly planting to characterize the growth orientation of leaves. Compared with VIs, it was less susceptible to soil and water effects and estimated rice LAI over the entire period with higher accuracy (Duan et al., 2019). Wavelet texture was used to eliminate the effect of rice spikes on the canopy during the heading period, enhancing the accuracy of estimating rice LAI after tasseling (Zhou et al., 2022). At present, the roles of different kinds of textures in the estimation of rice growth parameters and the appropriate scales are still unclear, and the mechanism of textures is challenging to interpret and requires further investigation.

In this study, rice was chosen as the research object, and the RGB and multispectral images of rice canopy were obtained at various time points, and spectral indices as well as multiscale texture information were extracted. The specific objectives are: (1) comparison of the ability of RGB-SI and MCA-SI to estimate single-period and multi-period LAI in rice; (2) comparison of the ability to estimate single-period and multi-period LAI in rice based on multi-scale texture features derived from RGB and MCA imagery; and (3) comparison of the ability of RF and MSR algorithms to incorporate SI and texture features for estimation of multi-period LAI in rice, and to perform multi-period LAI mapping in rice.

## 2 Materials and methods

### 2.1 Experimental design

**Figure 1:**
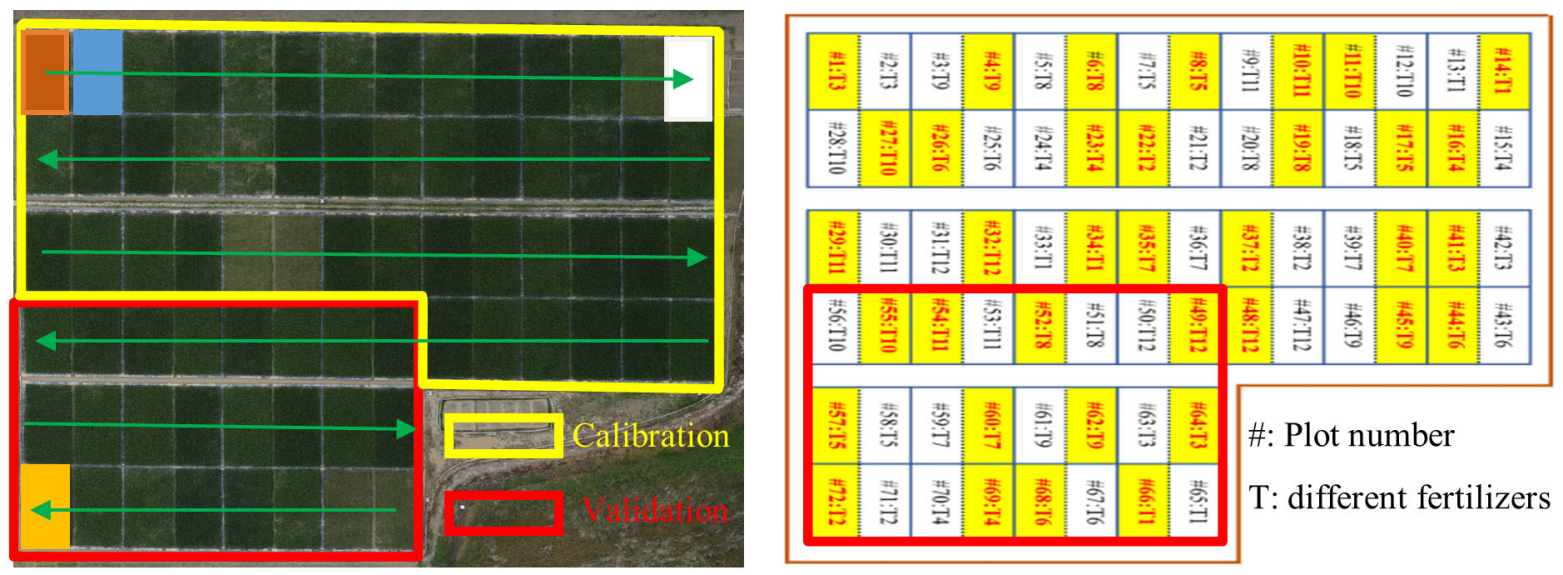
Experimental design and Distribution of rice plots.

The rice experiment was carried out from January to March 2023 at the South Propagation Base in Hainan Province (18°31′47″N, 110°3′35″E). A total of ten different types of fertilizers were designed in the experiment to simulate the growth condition of rice in reality, while no fertilizer (T1) and over-fertilization (T2) were used as control treatments. Two rice varieties, Fengyou No. 4 and Fengyou Xiang No. 1, which are widely planted locally, were selected as experimental subjects and randomly planted in 4m×8m plots and replicated three times, containing a total of 72 plots. The planting density was 288,000 plants/ha. Each fertilizer treatment was spaced by a black plastic film to prevent different fertilizer treatments from influencing each other. Of these, data from 48 plots were used for model construction, and the remaining 24 were used for model accuracy validation. The field management was performed by specialized personnel, and regular irrigation, drainage and weeding were carried out to ensure the normal growth of rice. The specific type of fertilizer was not the focus of this paper, so the formulation of the dosage forms involved in the experiment as well as the method of fertilizer application were not given. The control variables in this experiment were the fertilization treatments, which were kept consistent in terms of fertilization levels and field management such as drainage and irrigation, except for the different types of fertilizers used. The specific plots were distributed as shown in **Figure 1**.

### 2.2 UAV images and pre-processing

An autonomous modified octocopter UAV and a consumer-grade quadcopter UAV were used as remote sensing platforms to acquire rice remote sensing image data. Data were collected on 25 January (jointing stage), 13 February (booting stage), and 4 March (heading stage). The acquired image data include MCA multispectral and RGB images. There are twelve single-channel cameras (490, 520, 550, 570, 670, 680, 700, 720, 800, 850, 900, and 950 nm) integrated in the MCA camera (Tetracam, Inc., Chatsworth, CA, USA), each of which has independent CMOS (Complementary Metal-Oxide-Semiconductor) sensors, lenses, filters, and other image acquisition elements. Each CMOS sensor has a resolution of 1280*1024 pixels. DJI Phantom 4 Pro quadcopter drone was used for acquiring RGB visible light images. The flight path was set at 30 m altitude, 90% overlap in heading, 70% overlap in side direction, and equal time intervals for taking photos. The geometric processing of the UAV multispectral images acquired based on the MCA camera, including vignetting correction, band alignment, and aberration correction, was done in the PixelWrech2 (Tetracam, Inc., Chatsworth, CA, USA) software. The radiometric processing of MCA images consists mainly of radiometric calibration, which is referenced to (Luo et al., 2022b). Stitching and geometric correction of RGB images were done in Agisoft Photoscan Professional v1.4.5 software (Agisoft LLC, St. Petersburg, Russia).

In this paper, images of different spatial scales were obtained by nearest neighbor method resampling. Nearest neighbor resampling is a spatial interpolation method commonly used in image processing, computer vision, and geographic information systems (Brandsma and Können, 2006). The core idea of this approach is to estimate the data values at unknown locations by finding the nearest known data points on the basis of known data points. This method has been shown to be effective in feature extraction of UAV images of different resolutions. For each target pixel point, one or more pixel points closest to it were found for interpolation based on its position in the original image. The position of the target pixel point was mapped to the original image and then the closest pixel value was selected as the value of the target pixel point to complete the resampling of the image.

### 2.3 Leaf area index measurement

LAI was measured non-destructively by the LAI2200C canopy analyzer (LI-COR, Lincoln, Nebraska USA). LAI was calculated by measuring the above and below value (abbreviated as A and B values) of the rice canopy. According to the instructions in the LAI2200C manual, the instrument was placed in the middle of the two rows of rice plants when measuring the B value (ten B values in this work), and the B value measurements were evenly distributed throughout the plot. In addition, the instrument was covered with a 270°cover cap to avoid the influence of surveyors.

### 2.4 Feature extraction based on remote sensing images

#### 2.4.1 Spectral index

VI calculated from the combination of reflectance in two or more bands is one of the spectral features of UAV multispectral imagery and is the most commonly used image feature in rice LAI estimation. The calculation of spectral index (SI) based on RGB images is similar to the multispectral VIs, which is normalized by the DN values of different bands. The spectral indices (RGB-SI and MCA-SI) selected in this paper based on RGB and MCA images are shown in **Table 1**.

**Table 1:** The spectral indices selected in this paper based on RGB and multispectral images.

Types	Spectral indices	Formula	References
RGB-based spectral indices (RGB-SI)	R	DN values of R band	–
	G	DN values of G band	–
	B	DN values of B band	–
	r	R/(R + G + B)	(Woebbecke et al., 1995)
	g	G/(R + G + B)	(Woebbecke et al., 1995)
	b	B/(R + G + B)	(Woebbecke et al., 1995)
	ExR	1.4r - g	(Woebbecke et al., 1995)
	ExG	2g - r - b	(Woebbecke et al., 1995)
	ExB	1.4b - g	(Woebbecke et al., 1995)
	ExG-ExR	3g - 2.4r - b	(Woebbecke et al., 1995)
	VARI	(g - r)/(g + r - b)	(Gitelson et al., 2002)
	GRVI	(g - r)/(g + r)	(Zhu et al., 2023)
MCA-based spectral indices (MCA-SI)	Reflectance	Reflectance of 12 MCA bands	–
	NDVI	(R800nm - R670nm)/(R800nm + R670nm)	(Yan et al., 2022)
	NDRE	(R800nm - R720nm)/(R800nm + R720nm)	(Davidson et al., 2022)
	EVI2	2.5(R800nm - R670nm)/(R800nm + 2.4R670nm + 1)	(Mondal, 2011)
	GNDVI	(R800nm - R550nm)/(R800nm + R550nm)	(Rodríguez-López et al., 2020)
	CI_red edge_	R800nm/R720nm - 1	(Zheng et al., 2016)
	CI_green_	R800nm/R550nm - 1	(Zhang et al., 2015)
	VARI	(R550nm - R670nm)/(R550nm + R670nm)	(Schneider et al., 2008)
	OSAVI	1.16(R800nm - R670nm)/(R800nm + R670nm + 0.16)	(Steven, 1998)

#### 2.4.2 Gray level co-occurrence matrix

Gray-level Co-occurrence Matrix (GLCM) is a technique used to characterize image texture in image processing and analysis. It captures texture information by investigating the spatial correlation of the gray values of pixels in an image (Sethy et al., 2020). The basic concept of GLCM is to count the co-occurrence frequency of pixel pairs with specific gray values at a certain spatial distance and direction. A variety of texture features such as contrast and homogeneity can be extracted from GLCM, which are capable of rice growth characteristics such as roughness, uniformity, sharpness and complexity. In this paper, a total of eight parameters of mean (Mea), variance (Var), homogeneity (Hom), contrast (Con), dissimilarity (Dis), entropy (Ent), second moment (Sec), and correlation (Cor) in different bands were extracted as texture features.

### 2.5 Model construction and accuracy validation

Among all the samples were divided into training set and validation set according to 2:1 ratio, and two commonly used machine learning models, random forest (RF) and multiple stepwise regression (MSR), were used for LAI estimation (Lee and Lee, 2013; Shi et al., 2021). The model accuracy was assessed using the coefficient of determination (R^2^), root mean square error (RMSE), and mean absolute error (MAE). In the training set, the above metrics are R_cali_^2^, RMSEC, and MAEC. ln the validation set, the above metrics are R_vali_^2^, RMSEV, and MAEV.

## 3 Results

### 3.1 Correlation analysis of spectral indices and LAI for single and multiple periods

**Figure 2:**
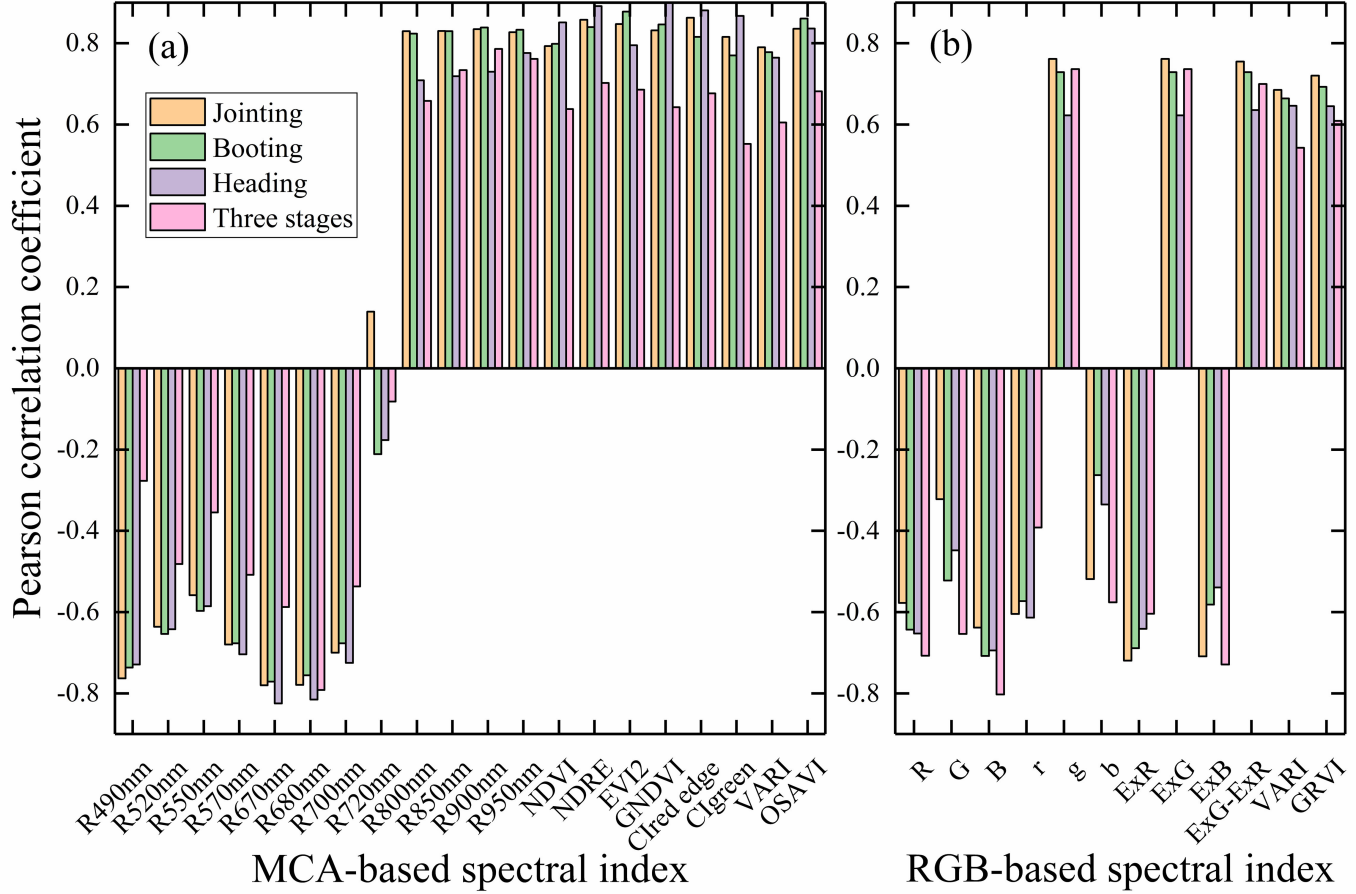
Correlation of spectral indices with LAI in single and multiple periods: **(A)** MCA-based spectral indices; **(B)** RGB-based spectral indices.

The spectral information of the rice canopy at different periods was extracted and correlated with the corresponding LAI using single-period and multi-period SI, and the results are shown in **Figure 2**. It can be seen that for MCA images, single-period SIs generally have high correlations with LAI. For example, the correlation between near-infrared (NIR) reflectance, different VIs and LAI exceeds 0.8. The correlation between multi-period SI and LAI decreases significantly and is generally below 0.6. For RGB images, the correlation between single-period and multi-period SI and LAI followed a similar trend to that of MCA-SI, with generally lower correlations for multiple periods than for single periods. However, the DN values of the original bands show the opposite trend, especially for the blue band, with a correlation with LAI of more than 0.8. Overall comparison shows that the correlation between MCA-SI and LAI is higher than that of RGB-SI.

### 3.2 Correlation analysis of multiscale GLCM and LAI for single and multiple periods

#### 3.2.1 RGB image-based GLCM

**Figure 3:**
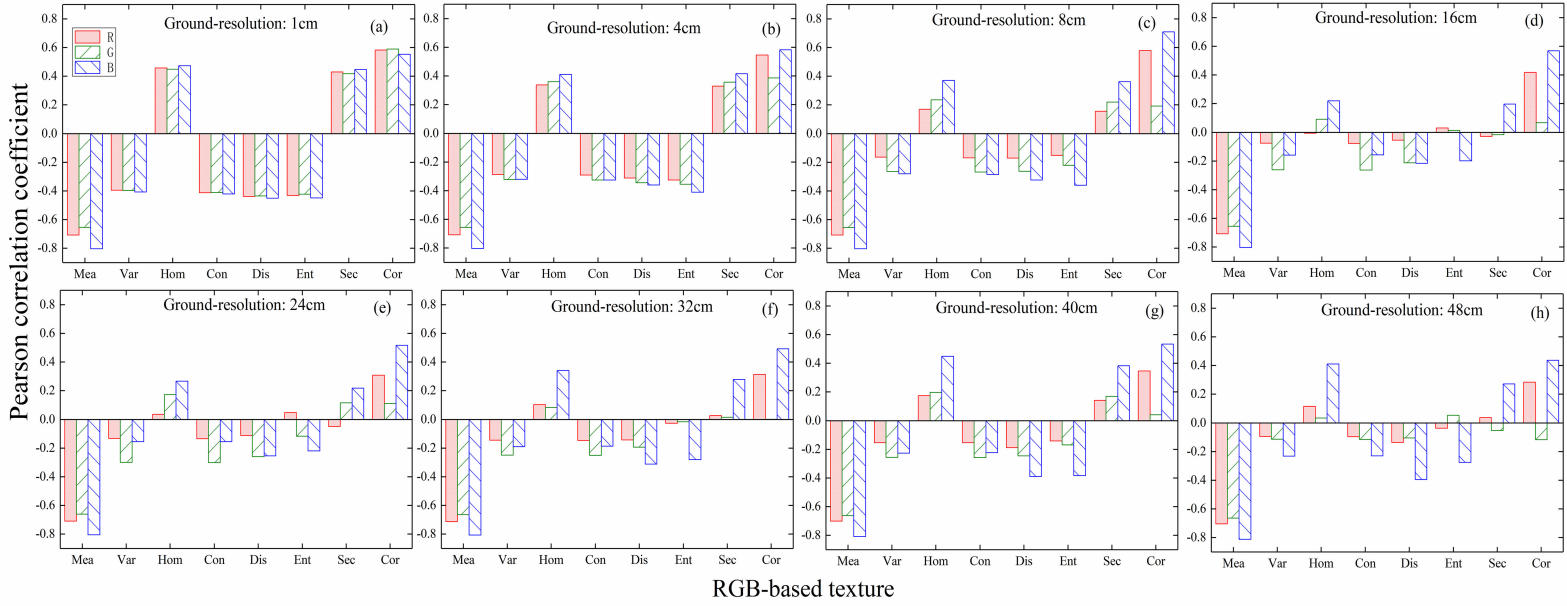
Correlation between texture and LAI in different bands of RGB images with different resolutions: **(A)** 1 cm; **(B)** 4 cm; **(C)** 8 cm; **(D)** 16 cm; **(E)** 24 cm; **(F)** 32 cm; **(G)** 40 cm; **(H)** 48 cm.

The results of the correlation between texture and LAI for different bands of RGB im-ages with different resolutions are shown in **Figure 3**. It can be found that among all the compared resolutions, the image texture at 1 cm resolution has the highest correlation with LAI, and the correlation between RGB-GLCM and LAI drops gradually as the resolution decreases. From different bands, among all the discussed resolution textures, those based on the blue band have the strongest correlation with LAI, and Mea shows the most out-standing performance, with a correlation with LAI close to 0.8. At 1-4 cm resolution, the GLCM in each band demonstrated a strong correlation with LAI. However, at resolutions below 16 cm, the correlation between texture and LAI in each band remained essentially unchanged, except for Mea, where the correlation was always very low. Therefore, the ultra-high resolution RGB image texture can well characterize the variation of rice LAI, and the coarse resolution will lead to a degradation of its performance.

#### 3.2.2 MCA image-based GLCM

**Figure 4:**
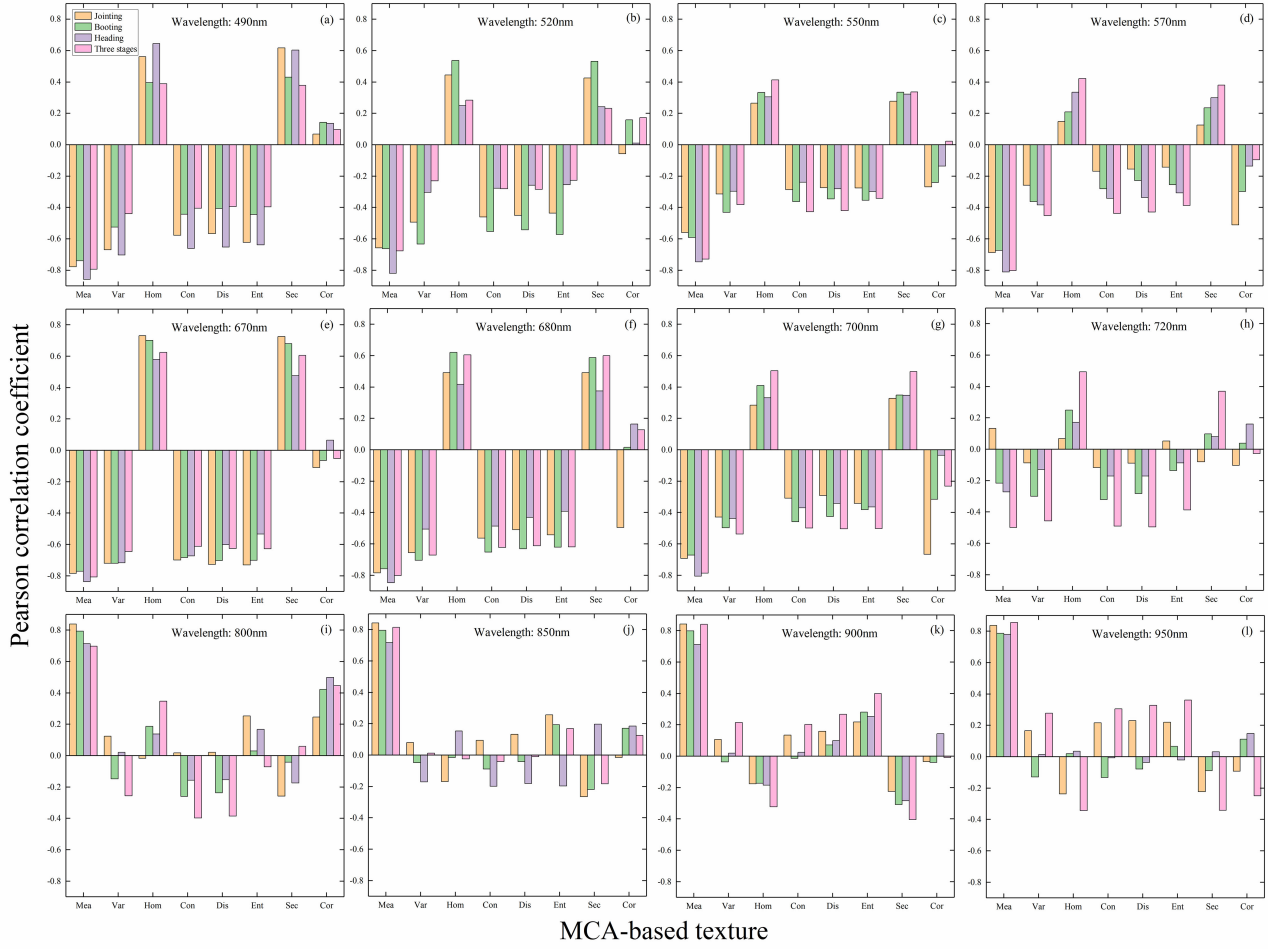
Correlation between texture and LAI in different bands of MCA images: **(A)** 490 nm; **(B)** 520 nm; **(C)** 550 nm; **(D)** 570 nm; **(E)** 670 nm; **(F)** 680 nm; **(G)** 700 nm; **(H)** 720 nm; **(I)** 800 nm; **(J)** 850 nm; **(K)** 900 nm; **(L)** 950 nm.

The acquired raw MCA images (with a resolution of 8 cm) were used to analyze the correlation of texture in different bands with LAI in a single period as well as in multiple periods, and the results are shown in **Figure 4**. Overall, it can be seen that the correlation between texture and LAI in each band of the single-period MCA image is stronger than that of the multi-period, with Mea showing the strongest correlation. Overall, it can be seen that the correlation between texture and LAI in each band of the single-period MCA image is stronger than that of the multi-period, with Mea showing the strongest correlation. In terms of different bands, the correlations between different textures based on the red bands (670 and 680 nm) and LAI are generally greater than 0.6 for both single-period and multi-period, with some higher than 0.8. Therefore, the 680 nm band of the MCA image was chosen to compute the texture for the subsequent studies in this paper.

**Figure 5:**
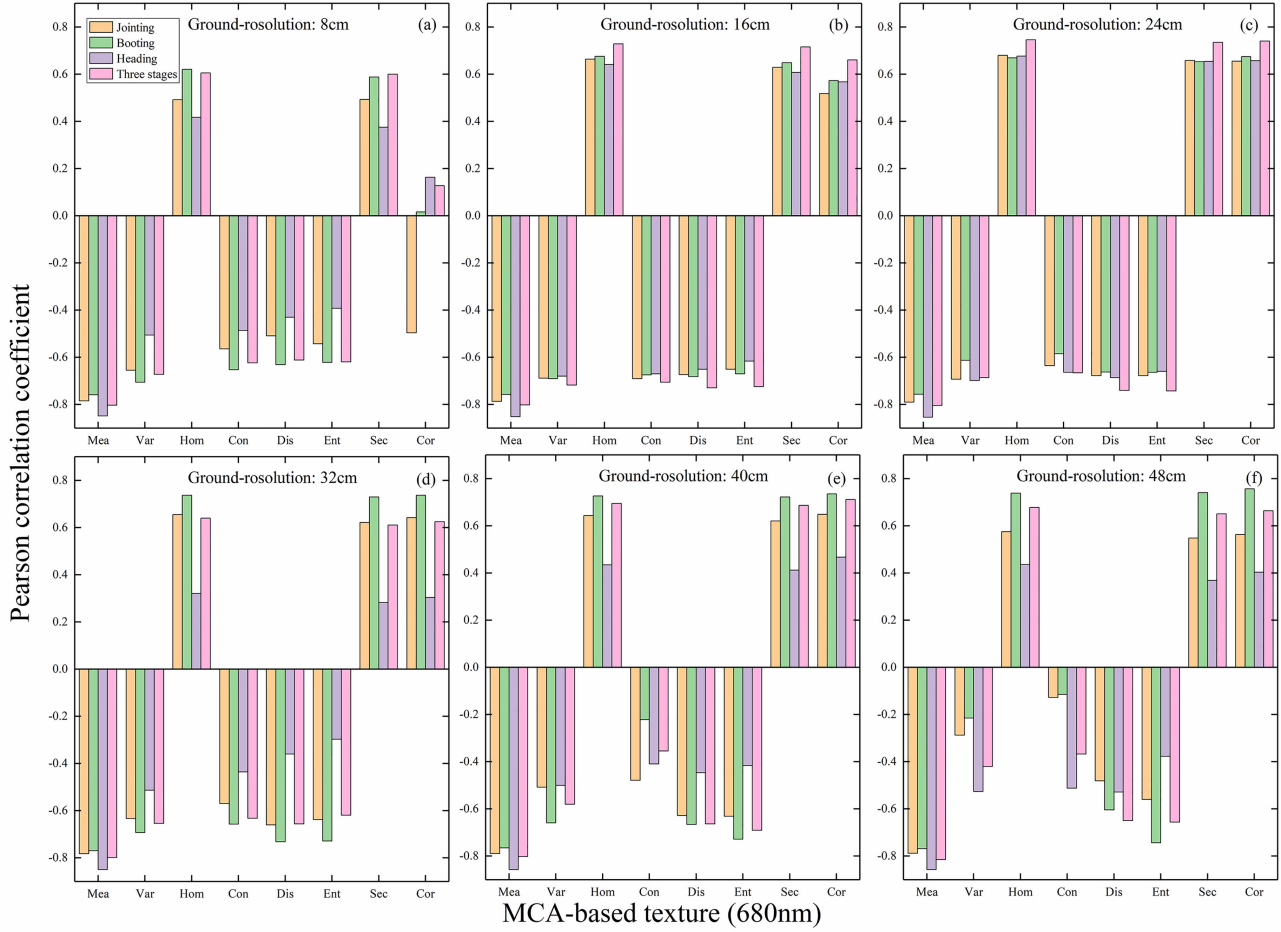
Correlation between texture and LAI in the 680 nm band for MCA images of different resolutions: **(A)** 8 cm; **(B)** 16 cm; **(C)** 24 cm; **(D)** 32 cm; **(E)** 40 cm; **(F)** 48 cm.

MCA images in the 680 nm band were utilized to resample to obtain other resolution images and to calculate the GLCM texture. The correlation between MCA image texture and LAI at different resolutions for single and multiple periods is shown in **Figure 5**. It can be observed that the correlation between texture and LAI based on each resolution remains basically stable with values around 0.7 in 8-40 cm resolution images. In addition, the correlation between multi-period texture and LAI is higher than that of single-period, and the texture information in this band shows great potential in the estimation of multi-period LAI in rice. The correlation of Var and Con with LAI decreases slightly in the 48 cm image. Overall, it appears that the correlation between texture and LAI is largely independent of resolution in the MCA 680 nm band images.

#### 3.2.3 Normalized difference GLCM

**Figure 6:**
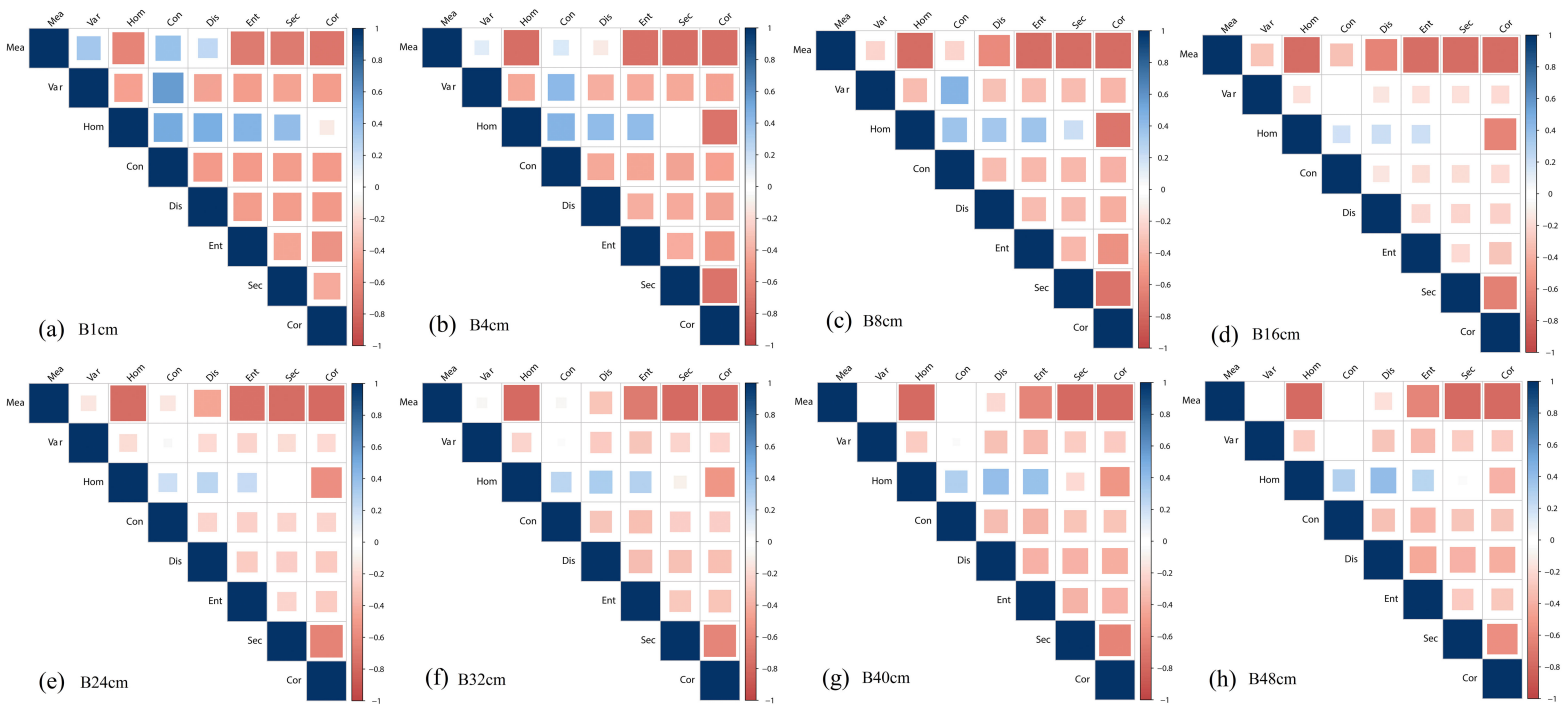
Correlation between NDT and LAI in the blue band of RGB images with different resolutions: **(A)** 1 cm; **(B)** 4 cm; **(C)** 8 cm; **(D)** 16 cm; **(E)** 24 cm; **(F)** 32 cm; **(G)** 40 cm; **(H)** 48 cm.

Similar to the calculation of normalized difference vegetation index (NDVI), different textures were normalized to enhance the ability of texture to characterize LAI. The correlation between normalized difference texture (NDT) and multi-period LAI is analyzed for images with different resolutions in the blue band of RGB images, and the results are shown in **Figure 6**. The larger the area of the squares and the closer the color is to blue or orange, the stronger the correlation is indicated. It can be seen that MeaHom, MeaEnt, MeaSec, MeaCor, VarCon have the strongest correlation with LAI in the original 1cm resolution image. As the resolution drops, the overall correlation between each NDT and LAI decreases, with only MeaHom, MeaSec, and MeaCor maintaining a stable correlation with LAI.

**Figure 7:**
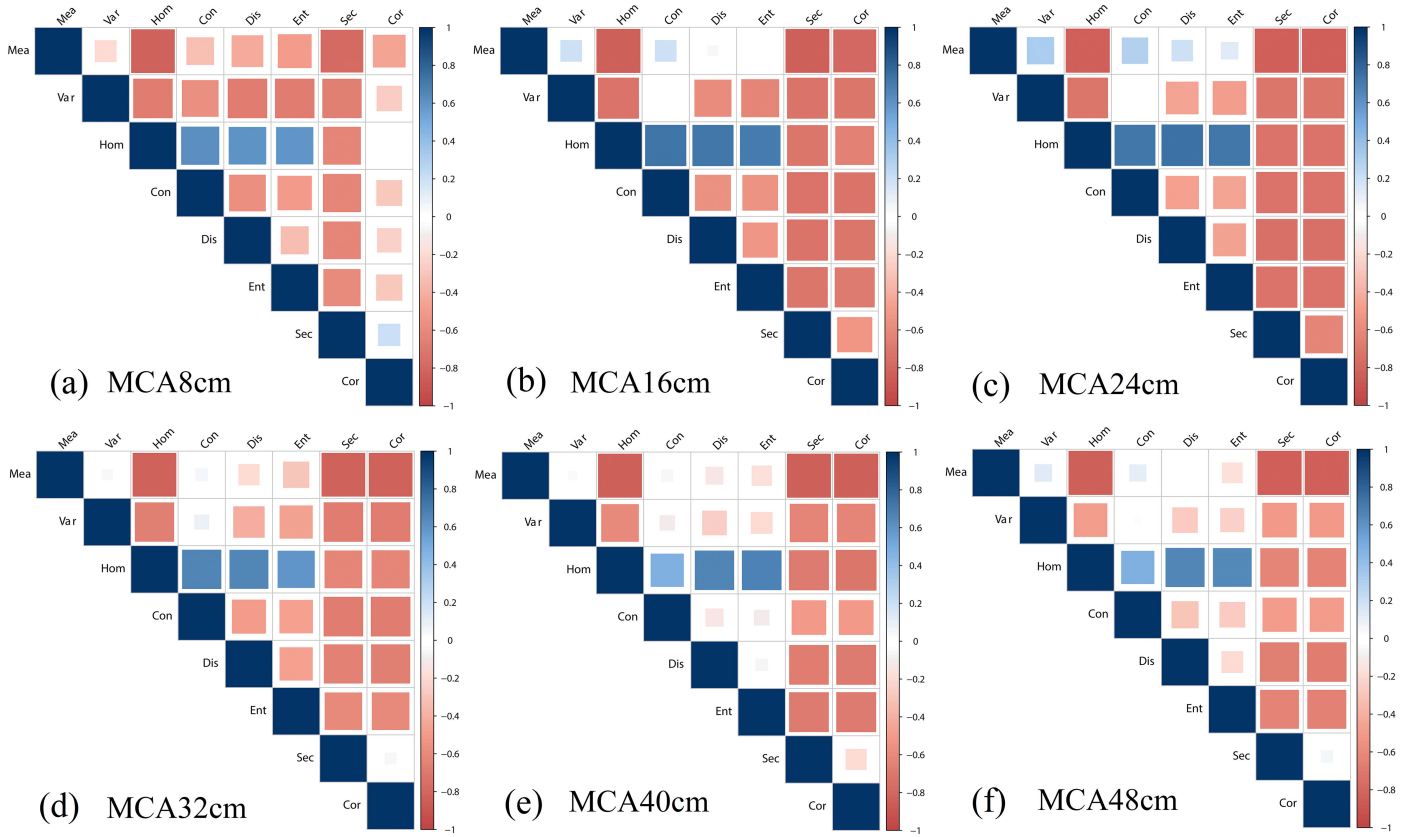
Correlation between NDT and LAI of MCA images (680 nm) with different resolutions: **(A)** 8 cm; **(B)** 16 cm; **(C)** 24 cm; **(D)** 32 cm; **(E)** 40 cm; **(F)** 48 cm.

Based on the 680 nm band of MCA images, the correlation between NDT and multi-period LAI was analyzed for different resolution images, and the results are shown in **Figure 7**. It can be noticed that there are more parameters that exhibit higher correlations with LAI for MCA-NDT compared to RGB-NDT, e.g., MeaHom, MeaSec, VarHom, VarDis, VarEent, VarSec, HomCon. With decreasing resolution, the correlation of most of the NDTs with LAI remains stable, especially for the combined NDTs of Sec, Cor, and Hom (two columns adjacent to the right and the third row).

#### 3.2.4 Estimating leaf area index using multiscale GLCM

The multi-period LAI estimation models based on all texture features including GLCM and NDT are constructed and validated using RF and MSR algorithms. The results of LAI estimation based on RGB texture features are shown in **Table 2**. It can be shown that although the LAI estimation model using RF algorithm has high training accuracy, the validation accuracy is obviously low. The accuracy of LAI estimation using RF decreases gradually as the resolution decreases. When estimating LAI using MSR, the difference between the validation and training accuracy is not significant, indicating that the model of MSR is more adaptable. When the resolution decreases, the accuracy of LAI estimation using MSR increases and then decreases, and reaches the highest accuracy at 4 cm resolution (R^2 =^ 0.7, RMSE = 0.65, MAE = 0.5). When using all textures to estimate LAI, the accuracy is not significantly improved and the model complexity is markedly increased. On the whole, it seems that the LAI estimation accuracy and model adaptation of MSR are higher than that of RF at the corresponding resolution.

**Table 2:** Models and accuracy of estimating multi-period LAI using multiscale texture features based on RGB images.

Resolution	dataset	RF			MSR			Equation
		R^2^	RMSE	MAE	R^2^	RMSE	MAE	
1cm	Cali	0.88	0.39	0.30	0.66	0.65	0.54	LAI=15.448-0.179*Mea1-9.274*meacor1
	Vali	0.65	0.62	0.49	0.67	0.67	0.52	
4cm	Cali	0.86	0.42	0.32	0.66	0.66	0.55	LAI=9.863-0.175*Mea4-12.465*homcor4
	Vali	0.62	0.64	0.50	0.70	0.65	0.50	
8cm	Cali	0.85	0.43	0.34	0.64	0.67	0.55	LAI=51.659-45.959*meaent8-45.078*homcor8
	Vali	0.61	0.65	0.51	0.63	0.73	0.56	
16cm	Cali	0.84	0.45	0.36	0.63	0.68	0.56	LAI=30.86-30.048*meacor16-64.9*seccor16
	Vali	0.59	0.67	0.52	0.62	0.72	0.56	
24cm	Cali	0.85	0.43	0.35	0.64	0.67	0.55	LAI=29.268-28.6*meacor24-92.39*seccor24
	Vali	0.54	0.71	0.55	0.61	0.73	0.57	
32cm	Cali	0.85	0.44	0.34	0.66	0.65	0.55	LAI=28.65-28.022*meacor32-119.2*seccor32
	Vali	0.49	0.75	0.58	0.64	0.73	0.58	
40cm	Cali	0.88	0.38	0.31	0.65	0.66	0.55	LAI=4.148-30.288*meacor40 + 28.014*cor40
	Vali	0.55	0.70	0.53	0.60	0.76	0.60	
48cm	Cali	0.85	0.43	0.34	0.64	0.67	0.56	LAI=-11.124-32.27*meacor48 + 44.35*Hom48
	Vali	0.53	0.71	0.55	0.63	0.74	0.59	
All	Cali	0.90	0.35	0.27	0.77	0.53	0.43	LAI=-38.476 + 74.826*Hom40 + 0.621*Var4-29.84*seccor4-0.192*Var1-33.566*meahom1
	Vali	0.64	0.62	0.49	0.70	0.60	0.49	

The results of LAI estimation using MCA image textures with different resolutions are shown in **Table 3**. It can be recognized that the MCA-based texture features estimate LAI with higher accuracy (R^2^ greater than 0.7) compared to RGB texture features. When using RF, the validation model accuracy is lower than the training accuracy. The LAI estimation accuracy remains essentially stable as the resolution decreases. When using MSR, the validation accuracy remains generally consistent as the resolution changes, and the accuracy is higher than that of RF. The accuracy of LAI estimation for RF and MSR was slightly improved when all textures are utilized as input variables, with MSR obtaining the highest accuracy at 16 cm resolution and all resolution texture inputs (R^2^ = 0.79, RMSE = 0.47, MAE = 0.38).

**Table 3:** Models and accuracy of estimating multi-period LAI using multiscale texture features based on MCA images.

Resolution	dataset	RF			MSR			Equation
		R^2^	RMSE	MAE	R^2^	RMSE	MAE	
8cm	Cali	0.86	0.41	0.31	0.70	0.61	0.50	LAI=5.935-6.795*meahom8-4.759*vardis8
	Vali	0.72	0.56	0.42	0.77	0.51	0.40	
16cm	Cali	0.87	0.40	0.29	0.70	0.61	0.49	LAI=7.879-7.626*meahom16
	Vali	0.74	0.53	0.38	0.79	0.49	0.38	
24cm	Cali	0.87	0.40	0.29	0.71	0.60	0.48	LAI=7.933-7.109*measec24
	Vali	0.73	0.54	0.41	0.76	0.51	0.39	
32cm	Cali	0.87	0.40	0.29	0.68	0.63	0.52	LAI=6.007-4.948*meacor32
	Vali	0.72	0.55	0.41	0.73	0.54	0.42	
40cm	Cali	0.86	0.41	0.30	0.71	0.61	0.50	LAI=5.759-4.722*meacor40
	Vali	0.73	0.54	0.41	0.74	0.53	0.41	
48cm	Cali	0.88	0.39	0.30	0.73	0.58	0.46	LAI=4.145-4.045*meacor48-1.746*discor48
	Vali	0.75	0.52	0.39	0.77	0.51	0.38	
All	Cali	0.90	0.35	0.26	0.75	0.56	0.45	LAI=5.568-5.823*measec24-2.108*discor48
	Vali	0.77	0.50	0.38	0.79	0.47	0.38	

### 3.3 Estimating leaf area index integrating SI and GLCM

RF and MSR were used to estimate multi-period rice LAI based on RGB and MCA images with SI, texture, and SI+texture as input variables, respectively, and the results are shown in **Table 4**. It can be noted that for training using the RF algorithm, the LAI estimation accuracy of MCA-based images is higher than that of RGB-based images when SI, texture, and SI+texture are used as input variables, respectively. The LAI estimation accuracy when modeled by the MSR algorithm is similar to that when modeled by the RF algorithm, i.e., the estimation accuracy based on MCA images is higher than that of RGB images. When estimating multi-period LAI, the accuracy of the model using texture features is higher than that using SI. In particular, the LAI estimation accuracy based on MSR-MCA and fusing SI and texture features is the highest (R_vali_^2 =^ 0.86, RMSEV=0.46, MAEV=0.35),and the validation accuracy is little different from the training accuracy with excellent stability.

**Table 4:** Models and accuracy of estimating multi-period LAI using spectral indices and multiscale texture features based on RGB and MCA images.

Type	Index	Equation	Rcali^2^	RMSEC	MAEC	Rvali^2^	RMSEV	MAEV
RF-RGB	SI	–	0.84	0.44	0.36	0.61	0.65	0.51
	Texture	–	0.90	0.35	0.27	0.64	0.62	0.49
	SI+ Texture	–	0.90	0.36	0.28	0.65	0.62	0.48
RF-MCA	SI	–	0.91	0.33	0.27	0.76	0.51	0.3.9
	Texture	–	0.90	0.35	0.26	0.77	0.50	0.38
	SI+ Texture	–	0.92	0.31	0.23	0.79	0.47	0.37
MSR-RGB	SI	LAI=6.085-0.042B-5.82ExB	0.66	0.65	0.54	0.68	0.70	0.53
	Texture	LAI=-74.8Hom40 + 0.6Var4-29.84seccor4-0.19Var1-33.6meahom1 + 38.5	0.77	0.53	0.43	0.70	0.60	0.49
	SI+ Texture	LAI=-48.92-0.2Mea48 + 56.34Hom40 + 0.02G	0.72	0.60	0.50	0.76	0.61	0.48
MSR-MCA	SI	LAI=-0.209 + 32.94*R900-24.29*R800	0.77	0.54	0.43	0.74	0.62	0.49
	Texture	LAI=5.568-5.8measec24-2.11discor48	0.75	0.56	045	0.79	0.47	0.38
	SI+ Texture	LAI=-3.2meacor48 + 18.68R900-4.85VARI+2.2	0.89	0.36	0.28	0.86	0.46	0.35

### 3.4 Leaf area index mapping of rice

**Figure 8:**
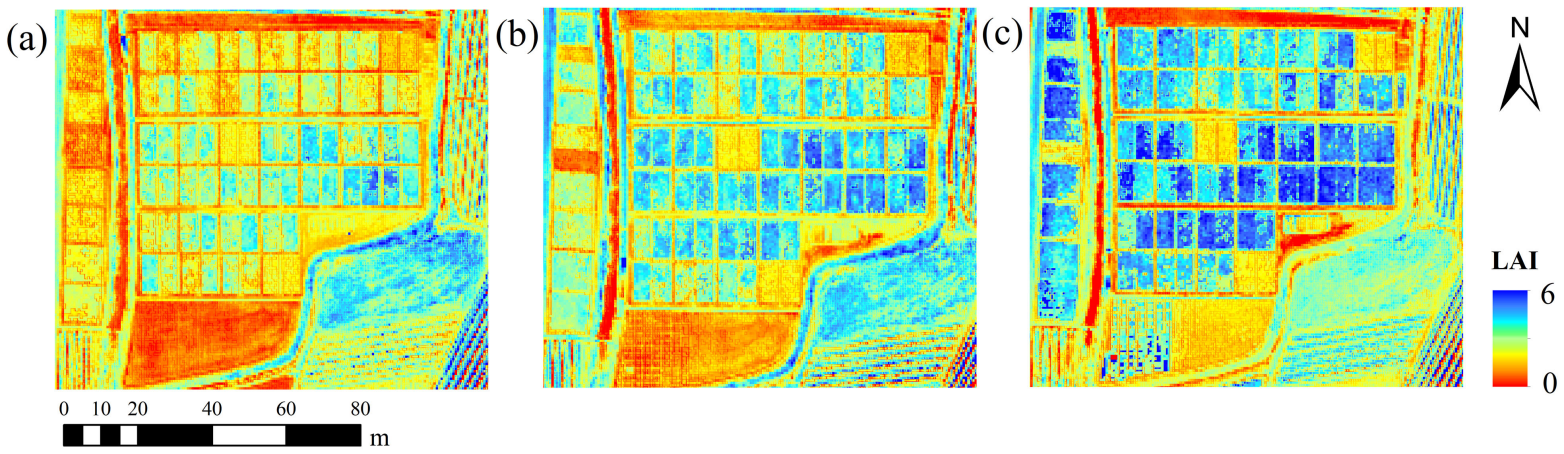
Spatial distribution of rice LAI in different periods: **(A)** jointing stage; **(B)** booting stage; **(C)** heading stage.

Based on the LAI estimation model of SI and NDT fusion constructed by MSR-MCA, the LAI of rice at different periods was mapped and the results are shown in **Figure 8**. We applied a pixel-by-pixel application of the corresponding image using LAI’s prediction formulas and then assigned different color representations. It can be observed that the spatial distribution map of LAI over time can well demonstrate the growth condition of rice. Under the spatial resolution of 48 cm, the growth differences within rice plots can be clearly observed, which is conducive to the optimization of field management and has important reference value for the large-scale application of high-resolution remote sensing images. A gradual increase in rice LAI over time could be obviously observed during the three different periods. Moreover, LAI displayed significant differences among 48 plots during a single period, which were influenced by the amount of fertilizer applied and the application method. In addition, for rice fields outside the study area, the model of this study can well reflect their growth conditions, indicating that the results of this study are generalizable.

## 4 Discussion

### 4.1 Trends in SI and texture with LAI

**Figure 9:**
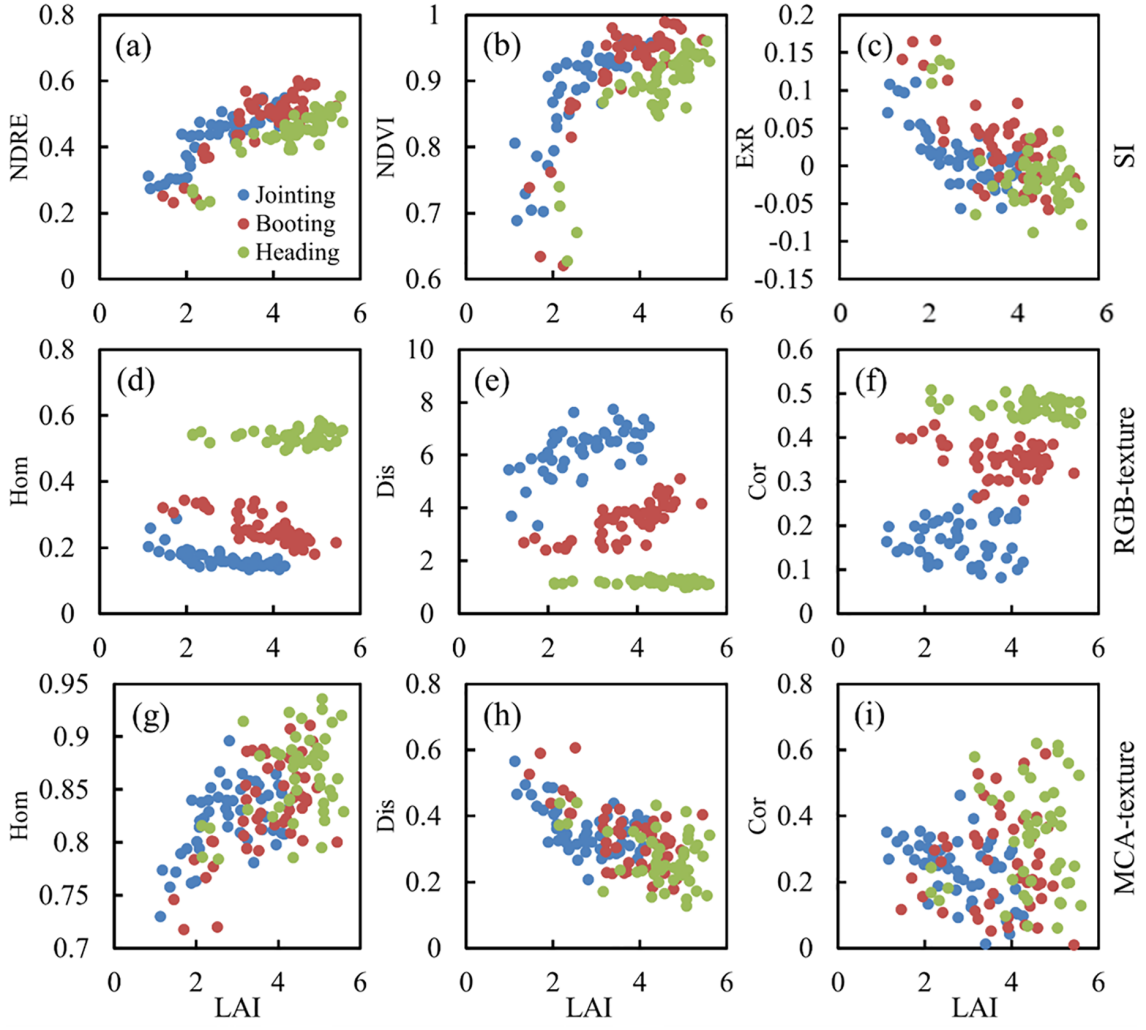
Trends in SI and texture with LAI at different stages: **(A)** NDRE; **(B)** NDVI; **(C)** ExR; **(D)** Hom-RGB; **(E)** Dis-RGB; **(F)** Cor-RGB; **(G)** Hom-MCA; **(H)** Dis-MCA; **(I)** Cor-MCA.

As can be seen from **Figure 2**, single-period SI has a strong correlation with LAI (the correlation coefficients between most of the SIs and LAI can exceed 0.8). However, for multiple periods, the correlation with LAI based on both RGB-SI and MCA-SI is significantly lower. **Figure 5** shows that the correlation between texture and multi-period LAI is significantly higher compared to a single period. Analysis of the trends of SI and texture with LAI (**Figure 9**) shows that NDRE, NDVI, and ExR can effectively characterize plot-scale LAI differences within a single period, but all indicators exhibit some level of saturation with increasing LAI across different periods. Hom, Dis, and Cor based on RGB imagery are strongly homogeneous within a single period, making it difficult to characterize LAI differences under different fertilizer treatments. The differences in these textures show large variations with changes in LAI at different periods. For the texture of MCA images, Hom, Dis, and Cor can not only characterize changes in rice LAI within a single period but also changes in LAI within different periods.

Spectral reflectance or VIs are often saturated in the estimation of crop LAI, biomass and other growth parameters (Yamaguchi et al., 2021). Since it is difficult for the spectral information of remote sensing images to penetrate deep into the canopy interior, it is difficult for VIs to characterize the true state of growth for denser crops, which results in the underestimation of growth parameters in the middle and late stages of crop growth (Li et al., 2023). Texture, as a variable that portrays distinct relationships between different pixels, can be used to add descriptive information about crop growth to the spectral information (Li et al., 2019; Yuan et al., 2023). During the tillering and jointing stages of rice, the field canopy consists mainly of rice and soil background. In the mid- to late-growth period, rice density gradually increases, and the field canopy is mainly composed of rice, which gradually increases in homogeneity and decreases in heterogeneity. Thus, these textural features can characterize rice LAI changes over time.

### 4.2 Role of multiscale texture in estimating rice LAI

In recent years, texture features based on different remote sensing images have been widely used to enhance the estimation of crop growth parameters (Zhang et al., 2022; Zhou et al., 2022). When using texture for the estimation of crop physiological and biochemical parameters, both image resolution and direction of texture computation are important variables that directly affect the relationship between texture and growth parameters. Yue et al. (2019) investigated the effect of image resolution on biomass estimation using texture and concluded that of all the resolutions studied (1-30 cm), the combination of textures at the highest (1 cm) and lowest (30 cm) resolutions was most suitable for estimating wheat biomass (Yue et al., 2019). Zheng et al. (2020) evaluated the effect of texture computation direction on estimating rice growth parameters and found that the texture computed perpendicular to the direction of the ridge was the most suitable for estimating rice nitrogen content (Zheng et al., 2020). Therefore, the texture calculation in this study adopts perpendicular to the monopoly direction as well.

**Figure 10:**
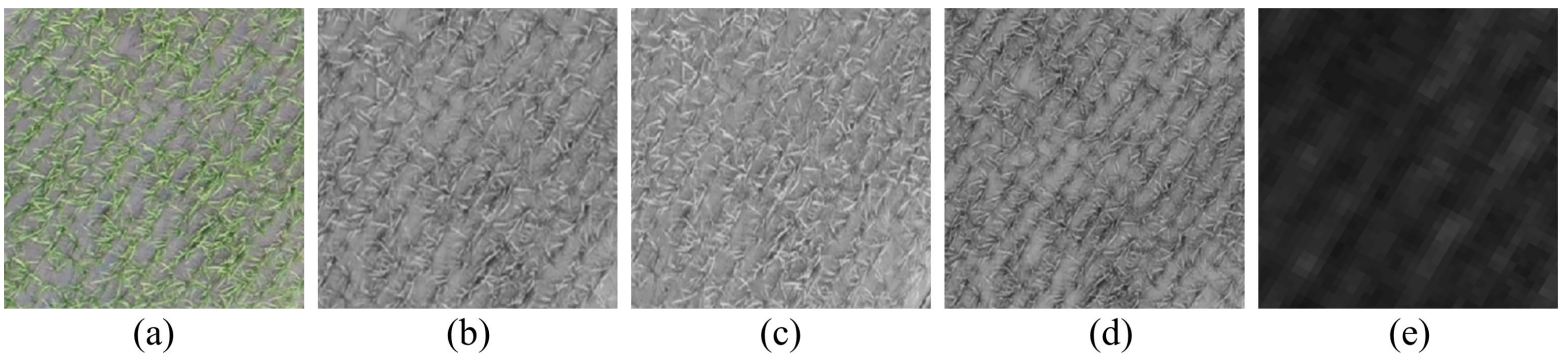
Images of rice fields in different bands: **(A)** RGB image; **(B)** red band of RGB image; **(C)** green band of RGB image; **(D)** blue band of RGB image; **(E)** 680 nm band of MCA image.

Changes in image resolution directly affect the canopy information reflected by the image. Yue et al. (2019) suggested that this is related to the differences in what the high-frequency information inside the image pixel represents at different resolutions. At centimeter resolution (1 cm), there are mostly pure pixels in the canopy image, when the high-frequency information reflects the growth of wheat, while at decimeter resolution (30 cm), there are mostly mixed pixels in the image, when the high-frequency information reflects the coverage of wheat, and thus the highest estimation accuracy can be obtained by combining the 1 cm and 30 cm textures (Yue et al., 2019). For the high-resolution image (RGB1cm, MCA8cm), the contrast between rice and soil background is shown in **Figure 10**. It can be observed that for the different bands of the RGB image, the blue band (**Figure 10D**) more accurately reflects the contrast relationship between the rice and the soil background. The red band (**Figure 10E**) of the MCA image more clearly illustrates the relationship between the soil background and the rice crop than the RGB image. Therefore, in **Figure 3**, the B-band texture of the RGB image shows a stronger correlation with LAI, and in **Figure 5**, the red-band texture derived from the MCA image exhibits a stronger correlation with LAI compared to the RGB image.

**Figure 11:**
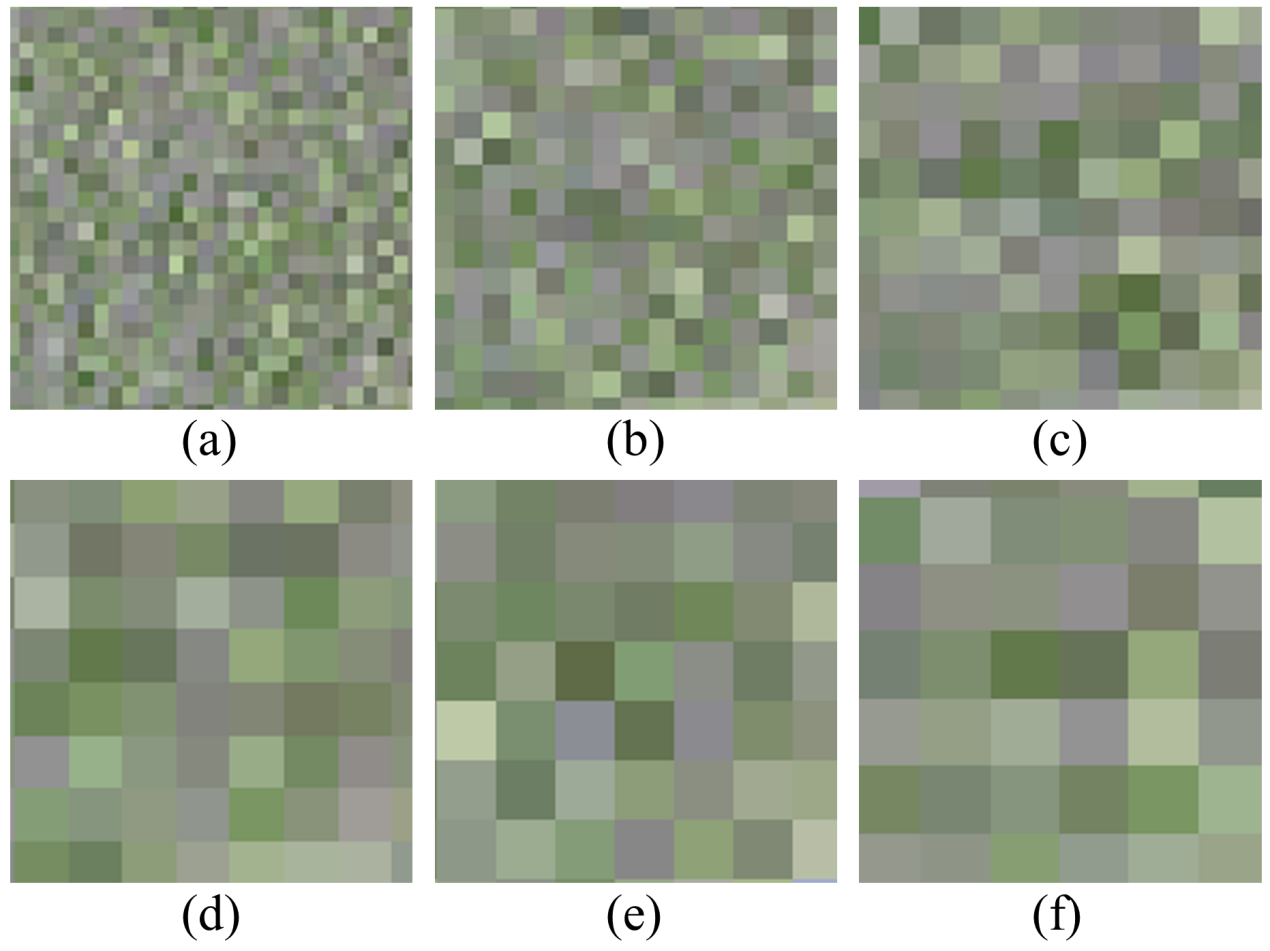
RGB images of rice fields at different resolutions: **(A)** 8 cm; **(B)** 16 cm; **(C)** 24 cm; **(D)** 32 cm; **(E)** 40 cm; **(F)** 48 cm.

For LAI estimation using images with different resolutions, the results in **Table 2** indicate that the accuracy of LAI estimation based on RGB image texture decreases as image resolution decreases. Conversely, the results in **Table 3** demonstrate that the accuracy of LAI estimation based on MCA image texture remains consistent despite variations in image resolution. This is due to the fact that the ultra-high resolution RGB image (**Figure 10A**) can clearly depict the relationship between the rice ridges and the soil background. However, after the resolution is reduced, as shown in **Figure 11**, it becomes challenging to distinguish the rice ridges in the image texture. At this point, there is no clear regularity in the contrast relationship between the soil and the rice. The resolution of the original MCA image is lower than that of the RGB image, so there is no difference in the relationship between rice and soil reflected as the resolution changes.

### 4.3 Advantages of fusing SI and texture features for estimating LAI in rice

**Figure 12:**
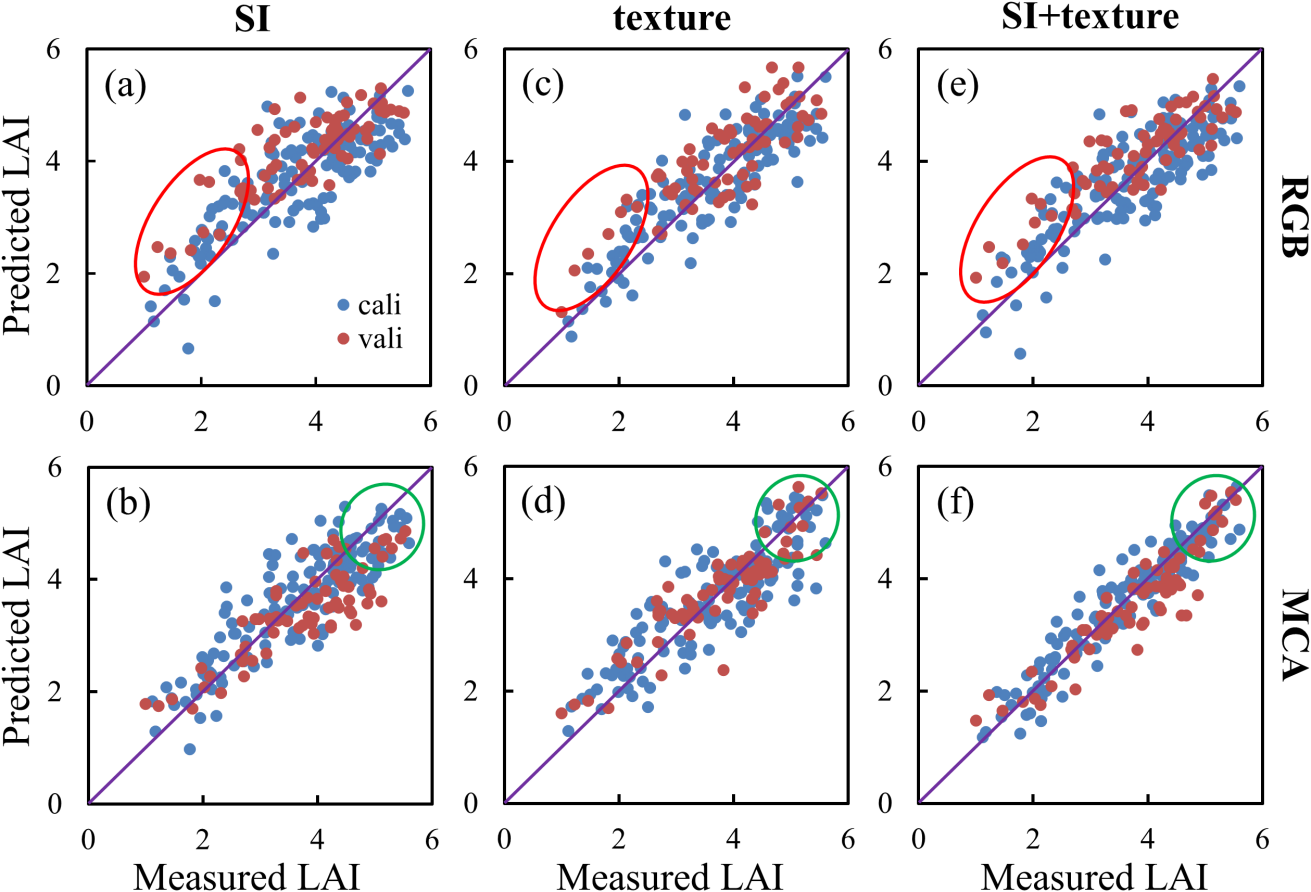
Comparison of predicted and measured LAI values for multiple periods: **(A)** SI-RGB; **(B)** texture-RGB; **(C)** SI+texture-RGB; **(D)** SI-MCA; **(E)** texture-MCA; **(F)** SI+texture-MCA.

Accurate crop canopy spectra reflect the interrelationship between crop growth dynamics and sunlight (Yu et al., 2023). The estimation results of estimating LAI using SI, texture, SI+texture, and based on the MSR algorithm, respectively, are shown in **Figure 12**. It can be seen that when estimating LAI based on RGB-SI, it is overestimated at low values of LAI (**Figure 12A**). After fusing texture and SI, the low LAI value is still overestimated (**Figure 10E**). The reason for this phenomenon may be that the SI calculation of RGB images does not strictly utilize canopy reflectance. This inaccurate spectral information makes it difficult to accurately characterize the variability of rice LAI. In contrast, MCA-SI did not overestimate or underestimate rice LAI at low values, which is consistent with the findings of related studies using multispectral or hyperspectral data (Liu et al., 2021). When using MCA-SI to estimate LAI, there was an obvious underestimation at high values of LAI (**Figure 12B**), which was caused by the saturation of multispectral SI in the mid- to late-stage of rice. The LAI continued to increase even when the SI reached its maximum value. On the other hand, MCA texture could have a beneficial auxiliary effect. As shown in **Figure 10D**, MCA texture can prevent the underestimation of high LAI values. After combining MCA-SI and texture features, both high and low LAI values can be predicted (**Figure 12F**).

In this study, MSR was utilized to fully fuse the SI and 0.48 m multispectral image texture information. The results of this research have significant potential for satellite-scale applications. With the continuous development of sensors, high-resolution satellite images are emerging (Yang et al., 2011). For example, the GeoEye-1 satellite has the capability to capture images at 0.41 m panchromatic resolution and 1.65 m multispectral resolution (Luo et al., 2017). Through image fusion, it can produce 0.41 m multispectral images. In addition, there are SuperView-1, WorldView-1, and WorldView-2 remote sensing images with approximately 0.5-meter resolution. All of these images have the potential for cross-platform application of the results of this study.

## 5 Conclusions

In this study, spectral indices and multiscale texture information obtained using RGB and MCA images are proved to be valuable for spatial and temporal prediction and mapping of rice LAI. The following conclusions can be obtained by comparing the accuracy of RF and MSR algorithms in fusing different information to estimate rice LAI.

1. The single-period SI based on RGB and MCA images showed a high correlation with LAI, but saturation in the mid- and late-growth stages of rice led to a significant decrease in the correlation between multi-period SI and LAI.
2. The texture features based on RGB images were influenced by resolution, while the 680 nm band texture of MCA images exhibited a strong correlation with multi-period rice LAI and remained unaffected by resolution. The ability to estimate rice LAI was enhanced by applying normalized difference processing to texture.
3. The accuracy of rice LAI estimation was significantly improved by fusing SI and texture features compared to single utilization of SI or texture features. Among the models based on fused information, MSR-based LAI estimation accuracy was the highest.

## Data Availability

The original contributions presented in the study are included in the article/supplementary material. Further inquiries can be directed to the corresponding author.
